# No evidence for colonization of oral bacteria in the distal gut in healthy adults

**DOI:** 10.1073/pnas.2114152118

**Published:** 2021-10-05

**Authors:** Armin Rashidi, Maryam Ebadi, Daniel J. Weisdorf, Massimo Costalonga, Christopher Staley

**Affiliations:** ^a^Department of Medicine, University of Minnesota, Minneapolis, MN 55455;; ^b^School of Dentistry, University of Minnesota, Minneapolis, MN 55455;; ^c^Department of Surgery, University of Minnesota, Minneapolis, MN 55455

**Keywords:** gut microbiota, oral microbiota, *Dialister invisus*

## Abstract

The microbial communities in the mouth and colon are anatomically connected via the saliva. However, the extent to which oral microbes reach and successfully colonize the distal gut has been debated. To resolve this long-standing controversy, we used exact amplicon sequence variants generated from concurrently collected saliva/stool microbiota in 66 healthy adults from two countries to show that, with one exception (*Dialister invisus*), the two niches are completely distinct. Thus, there is no evidence for colonization of oral bacteria in the distal gut. This defines the healthy state to which pathological states could be compared. Finding the same bacteria in the mouth and stool may warrant clinical investigation for an underlying pathology.

The ingestion of food and ∼1 L to 1.5 L of saliva every day creates a shower of ∼10^11^ oral bacterial cells to the intestinal tract (1), thus establishing an anatomic connection between the two microbial habitats. To colonize the distal gut, oral bacteria need to 1) arrive in the gut alive, 2) adapt to the physicochemical characteristics of the new habitat (e.g., low oxygen pressure, toxins present in fecal content), and 3) avoid elimination by the gut microbiota. Several barriers exist along the way, including gastric acid, bile salts, mucosal immunoglobulins, and antimicrobial peptides. The extent of colonization of oral bacteria in the distal gut has been debated, with some studies suggesting a substantial overlap between the two niches (2, 3), while others finding minimal overlap (4–6). This is an important knowledge gap, with potential clinical implications. As an example, some patients with colorectal cancer have identical strains of *Fusobacterium nucleatum* in their colon and mouth, suggesting ectopic colonization and a potential carcinogenic effect (7). Using exact amplicon sequence variants (ASVs) generated from concurrently collected saliva/stool microbiota in 66 healthy adults from two countries, we find that the two niches are almost completely distinct, with no evidence for colonization of oral bacteria in the distal gut. Compared to operational taxonomic units, which are clusters of sequence reads differing by less than a fixed dissimilarity threshold (typically 1 to 3%), ASVs are resolved at the level of single nucleotide differences and can be used to identify species, subspecies, and strains (8).

## Methods

Although several niches exist in the oral cavity, we chose saliva because it contains microbes from the other oral niches (9) and is the main vehicle via which the oral microbiome may reach the distal gut. We used publicly available data (National Center for Biotechnology Information [NCBI] BioProject SRP057504) containing stool and salivary samples collected concurrently from 66 healthy adults from the United Kingdom and Sweden in a randomized clinical trial (10). The trial included samples at baseline and after exposure to a single dose of antibiotic vs. placebo. Baseline samples and their raw single-end reads from 454 pyrosequencing of the V5 to V7 hypervariable segments of the 16S ribosomal RNA gene were downloaded and analyzed.

Adaptor trimming was done in Quantitative Insights Into Microbial Ecology (QIIME) 2 using SHI7 (11), and the resulting demultiplexed fastq files were used as input to Divisive Amplicon Denoising Algorithm (DADA)2 (12). ASVs were inferred using the dada2 package v1.18.0 in R 3.4 (R Foundation for Statistical Computing). DADA2 implements a de novo process which distinguishes biological sequences from errors based partly on the former’s higher expected rate of occurrence (8). Filtering, dereplication, denoising, merging, and chimera removal were done using DADA2 default parameters (*SI Appendix*). Single-end reads were truncated at 300 bp, yielding a quality score of >30 in >90% of the reads. Because of the 454 pyrosequencing platform associated with high rates of indels, a homopolymer gap penalty of −1 and a band size of 32 were used. The pseudopooling processing mode was used to allow information sharing across samples, improving sensitivity to possibly rare ASVs. Taxonomic assignment was done according to the naive Bayesian classifier method implemented within DADA2 and using the SILVA nonredundant v138.1 training set (13). The minimum bootstrapping support (parameter minBoot) required to return a taxonomic classification was set to 80. Species assignment was performed by exact string matching against the SILVA species assignment training database (13). The ASV table was merged with relevant metadata into a phyloseq object for downstream analysis in R (*SI Appendix*). We measured niche−niche overlap using the Jaccard index (14), defined as the size of the intersection divided by the size of the union of the two samples. Presence/absence data are used to determine this index. Jaccard distance, calculated by subtracting the index from one, indicates the extent of separation of the two samples.

## Results and Discussion

**Fig. 1.**
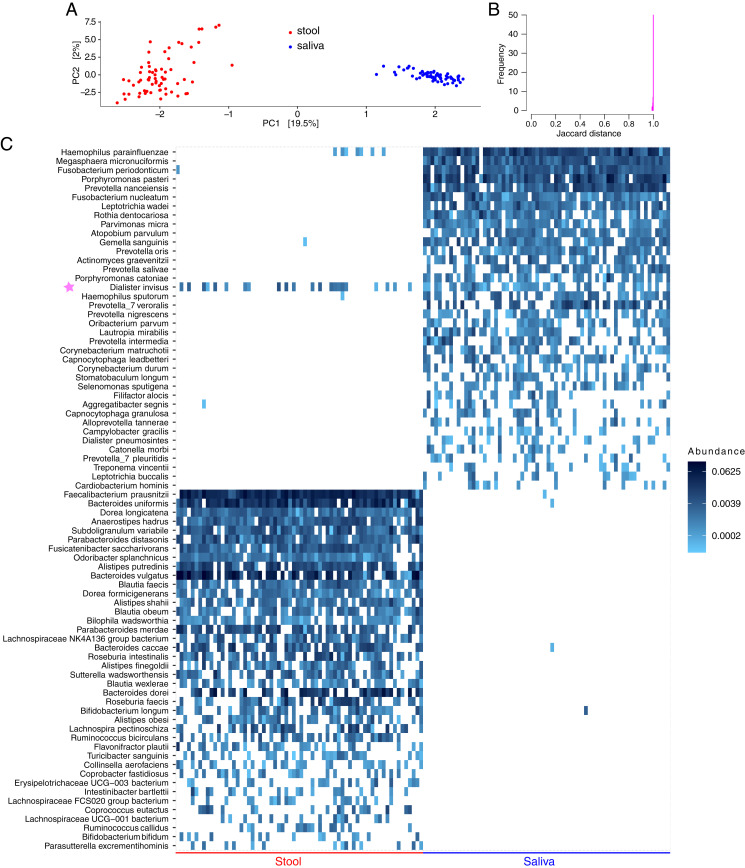
Comparison of salivary vs. colonic microbiota in healthy adults. Data come from a public database of concurrently collected stool and salivary samples from 66 healthy adults in two countries. Raw 454 pyrosequencing reads obtained from the V5 to V7 hypervariable segments of the 16S ribosomal RNA gene were analyzed. Exact ASVs were inferred using DADA2 and mapped to the species level. (*A*) PCA using centered log-ratio ASV abundances. Each point shows a sample. Numbers in square brackets indicate the fraction of total microbiota variation explained by the corresponding axis. (*B*) Histogram of Jaccard distance between concurrent stool and salivary samples for all subjects. (*C*) Heatmap of the 50 most prevalent species in each habitat showing near-complete separation of the distal gut vs. salivary microbiota. Each column represents a sample, each row represents a species (corresponding to one or more ASVs), and color coding represents relative abundances. Rows for each niche are arranged by taxon prevalence among samples. The only overlap between the two habitats was *D. invisus* (with one ASV), indicated by a purple star. Although there also appeared to be a small overlap in *Haemophilus parainfluenzae*, this species included five ASVs, detected in zero, one, two, two, and two samples, respectively, indicating minimal to no overlap. The plot_heatmap function in R (*SI Appendix*) with taxa.label=”Species” shows only the species part of each taxon (e.g., *parainfluenzae*), and, with taxa.label=”Genus”, only the genus part (e.g., *Haemophilus*). Thus, plot_heatmap was run twice, once using taxa.label=”Species” and once using taxa.label=”Genus”. Taxon names from the two plots were combined in Inkscape to generate the full name for each taxon (e.g., *Haemophilus parainfluenzae*).

Sample depth ranged between 2,793 and 22,414, with a mean (median) of 9,146 (8,698) reads per sample. After filtering, 5,494 ASVs (66 stool and 66 saliva samples from 66 subjects) were retained. In principal component analysis (PCA) using centered log-ratio ASV abundances, salivary and stool microbiota distinctly segregated (Fig. 1*A*), indicating different compositions. Similarly, using ASV presence/absence data, there was almost no overlap between salivary and stool microbiomes, with a mean (range) Jaccard distance of 0.998 (0.989 to 1.000) across all individuals (Fig. 1*B*). A microbiota heatmap confirmed these findings, with the only exception being a *Dialister invisus* ASV, with significant overlap between the two niches (Fig. 1*C*). A nucleotide basic local alignment search tool (BLASTn) search mapped this ASV to *D. invisus* type strain (DSM 15470) and *D. invisus* strain JCM 17566 (100% identity; e-value 4 × 10^−150^). This ASV was present in the saliva of 39 (59.1%) subjects, stool of 22 (33.3%) subjects, and both saliva and stool of 16 (24.2%) subjects. Using the first two percentages as surrogates for the probability of finding this ASV in the saliva and stool in the healthy adult population, the expected probability of finding the same ASV in both habitats, without a need to assume a connection between the two, would be 59.1% × 33.3% = 19.7%, only slightly lower than the observed 24.2% rate of co-occurrence. The relative abundance of this ASV was higher in stool than saliva in 8 of the 16 subjects with co-occurrence, further arguing against ectopic colonization in most cases.

*D. invisus* is a predominantly oral bacteria (15) that is also found in the distal gut, but with little transcriptional activity (5). Although the origin and functional significance of this bacteria in the distal gut are unknown, its frequency of co-occurrence in saliva and stool was comparable to its probabilistically calculated expected rate without a need to invoke oral−gut connectivity. Five previous studies evaluated the overlap between oral and distal gut microbiomes. The first study used genus-level data (3), an approach that is unable to ascertain co-occurrence due to insufficient taxonomic resolution. The second study used reference-based microbial single nucleotide variants for strain identification (2). However, the computational pipeline used in this study (metaSNV) makes the assumption that there is one dominant strain per species (16). This assumption is problematic when studying the human microbiota which is often a mixture of closely related strains of the same species. Using a single reference sequence to represent a species during mapping can drastically overestimate the overlap between samples by neglecting strain-level differences. The third study used shotgun sequencing and found a small overlap between saliva and distal gut microbiomes (eight species/strains) (5). The only abundant gut bacterium with appreciable levels in the oral communities was *D. invisus*. The fourth study used oligotyping, a supervised computational method for partitioning sequence data based on highly polymorphic nucleotide positions within otherwise identical 16S amplicons (6). The overlap between oral and stool microbiota in this study was characterized by one oligotype, matching *D. invisus*. The fifth study used metagenome-assembled genomes and found only two genomes—both mapped to *D. invisus*—that were shared between the two habitats (4).

In conclusion, oral and distal gut microbiomes in healthy adults are highly distinct, with *D. invisus* being a notable exception of unclear significance. We found no evidence for colonization of oral bacteria in the distal gut. This extreme separation of the two habitats may diminish in disease states in which the chemical or immunological barriers or the colonization resistance of the gut are compromised. Finding the same bacteria in the oral cavity and stool may warrant clinical investigation for an underlying pathology.

## Data Availability

Anonymized raw sequence reads have been deposited in NCBI BioProject (SRP057504).
